# What determinants of COVID-19 vaccine hesitancy among Chinese nursing students? A cross-sectional study

**DOI:** 10.3389/fpubh.2024.1432225

**Published:** 2024-08-20

**Authors:** Xin Wang, Ming Liu, Yuanzhen Li, Xiaoxiao Mei, Shuting Liao, Qingqing Liang, Yachen Liu

**Affiliations:** ^1^Faculty of Health Sciences and Sports, Macao Polytechnic University, Macao, Macao SAR, China; ^2^Peking University Health Science Center-Macao Polytechnic University Nursing Academy, Macao Polytechnic University, Macao, Macao SAR, China; ^3^School of Nursing, Wannan Medical College, Wuhu, China; ^4^School of Nursing, Hong Kong Polytechnic University, Hong Kong, China

**Keywords:** attitudes, COVID-19, vaccine, nursing students, vaccine hesitancy

## Abstract

**Background:**

The coronavirus disease 2019 (COVID-19) continues to threaten human health, and health professionals, including nursing students, usually work in healthcare frontiers with a high risk of infection. Vaccination is currently one of the most effective preventive measures. This study aimed to explore the determinants of COVID-19 vaccine hesitancy in nursing students.

**Methods:**

In November 2022, a sample of undergraduate nursing students was recruited from several medical schools in Anhui Province, China, and an online cross-sectional survey was conducted using the questionnaire star platform (Wenjuanxin). A Chi-square test was used to explore vaccine hesitancy among nursing students with different social demographic characteristics and vaccine attitudes. Binary logistic regression analysis was then used to determine the influence factors of vaccine hesitancy among nursing students.

**Results:**

A total of 1,090 valid samples were collected in this study. Of these, 27.06% (295) of nursing students reported COVID-19 vaccine hesitancy. The results showed “the need to go out of town recently” (OR = 0.670), “very confident that the outbreak could be controlled sustainably” (OR = 0.393), “feeling at risk of infection” (OR = 0.658), “not being worried/being generally worried about the vaccine's safety” (OR = 0.226 and OR = 0.686, respectively), and “not being worried about the vaccine's effectiveness” (OR = 0.411). These five factors are protective factors associated with COVID-19 vaccine hesitancy in nursing students. The factors “considering the country completely safe from an outbreak” (OR = 3.436), “considering themselves safe because others are vaccinated” (OR = 2.239), and “Agreeing that other protective measures can be relaxed after vaccination with the COVID-19 vaccine” (OR = 2.007) are risk factors associated with COVID-19 vaccine hesitancy among nursing students (*P* < 0.05).

**Conclusion:**

Overall, relatively few nursing students had COVID-19 vaccine hesitancy. Schools and relevant institutions still need to actively guide them to improve their confidence in the COVID-19 vaccine, strengthen the prevention and control measures of the epidemic, and improve their awareness of the crisis to improve the vaccination rate to reduce the COVID-19 vaccine hesitancy in nursing students.

## 1 Introduction

The outbreak of coronavirus disease 2019 (COVID-19) in 2020–2022 seems to be behind us, however, in fact, it still threatens human health worldwide and causes severe social and medical resources burden. World Health Organization (WHO) reported on April 11, 2024, that there are still thousands of deaths per week from COVID-19, and the number of deaths per week is distributed in a wave pattern (1). The report emphasizes that COVID-19 continues to threaten the health of our population, and vaccination with COVID-19 is the most effective preventive measure at present. Vaccination with COVID-19 can effectively prevent the disease from becoming severe and reduce mortality. Furthermore, given the changing nature of outbreaks and the potential for mutation and waning immunity, regular COVID-19 vaccination is necessary to counter the threat posed by long-term COVID-19 (2).

However, with the effective control of infectious diseases by vaccination, people turn to worry about the safety and necessity of vaccination, resulting in obvious vaccine hesitancy (3). Vaccine hesitancy refers to delaying the acceptance or refusal of vaccination despite the availability of vaccination services. Vaccine hesitancy is complex and context-specific, varying across time, place, and vaccines. It is influenced by factors such as complacency, convenience, and confidence (4). Individuals who are vaccination hesitant fall between the vaccine-willing and vaccine-refusing categories, including those who refuse certain vaccines but receive others, those who delay vaccination, and those who are willing to be vaccinated but have concerns (5). According to the 3Cs model proposed by WHO (4), vaccine hesitancy is influenced by confidence, convenience, and complacency factors. Confidence represents trust in vaccine safety, effectiveness, and health system capabilities. Complacency means a low perception of disease risk; vaccination was not considered necessary. Convenience represents the convenience of the vaccination environment and the affordability and availability of vaccines (4). Social contexts also influence vaccine hesitancy, including vaccination history, economics, culture, ecology, health systems, and politics (3). In addition, individual or group views and beliefs about vaccines and attitudes toward vaccination influence individual vaccine hesitancy (6).

In 2019, the WHO listed vaccine hesitancy as one of the world's top ten health threats (7). Vaccine hesitancy is thought to cause lower vaccine coverage and increased risk of a vaccine preventing disease outbreaks and epidemics (4). Studies have shown that when COVID-19 vaccination rates reach 67% of the population, the incidence of COVID-19 decreases (8). However, the overall incidence of COVID-19 vaccine hesitancy in different populations at this stage is 31.1–84.6% (9). How to alleviate public vaccine hesitancy and improve the vaccination rate of COVID-19 has become a new challenge for COVID-19 epidemic prevention.

Front line healthcare workers are considered one of the groups at highest risk of exposure to COVID-19 infection, and with evidence showing that the percentage of healthcare workers testing positive for COVID-19 was 51.7%, healthcare workers are at great risk and challenge during outbreaks (10). As future healthcare workers, nursing students need to complete clinical placements despite the ongoing global epidemic. Moreover, nursing students are at a relatively high risk of infection due to a lack of knowledge about infectious diseases, less clinical experience, and inadequate protective measures (11, 12). To ensure their health, vaccination of COVID-19 vaccine is essential for nursing students. Besides, as providers and educators of health care, nursing students' attitudes to vaccination will affect their health and the attitude of other people around them toward vaccination (13), they have a key role to play in promoting the delivery of vaccinations. Several studies have shown that the medical staff's knowledge and attitude to vaccines are essential determinants of patient acceptance of vaccines (14, 15). Therefore, it is important to understand the determinants of COVID-19 vaccine hesitancy in nursing students and to increase vaccine knowledge and vaccination rates among nursing students.

Vaccine hesitancy is also a psychological state of refusing or resisting vaccination, including behavioral vaccination, but still with doubt or suspicion (16). Although most medical personnel and medical students in China have taken the initiative to vaccinate against COVID-19, this does not mean that they do not have vaccine hesitancy. Negative vaccination mentality and vaccination behaviors may influence individual attitudes toward vaccines and future vaccinations (17). Scholars from several different countries have studied the current status of COVID-19 vaccine hesitancy among nursing students. A cross-sectional study conducted in the United States revealed that 31.1% of nursing students exhibited hesitancy about vaccination, while 16.4% expressed a lack of intention to be immunized. Influence factors of vaccine hesitancy include a positive attitude, concerns about safety, consulting social media, and race (18). A study of 1,068 medical students in India revealed that 10.6% of individuals exhibited vaccine hesitancy. Factors such as concerns about vaccine safety and effectiveness, lack of awareness of vaccination qualifications, and lack of trust in government agencies were identified as influence factors of vaccine hesitancy (19). However, most existing studies on vaccine hesitancy have tended to conceptualize it in overly simplistic terms, categorizing into “acceptance,” “rejection,” and “uncertainty.” Vaccine hesitancy is a complex psychological state. As such, these studies should exercise more caution and nuance when interpreting and characterizing the phenomenon of vaccination hesitancy.

Reducing nursing students' COVID-19 vaccine hesitancy is recognized as an important strategy for promoting public confidence in vaccines and preventing disease. With the continuation of the COVID-19 epidemic, there is an increased need for future interventions to prevent outbreaks. Governments have emphasized leveraging behavioral science insights to identify public concerns and develop response strategies to promote behavioral interventions for COVID-19 prevention (20). Increasing the intention of nursing students to receive the COVID-19 vaccine is recognized as an important strategy to prevent infection and build public confidence in vaccination (12). However, the evidence shows that worldwide, the percentage of medical students hesitant about the COVID-19 vaccine ranged from 3.9 to 66.2%, and only concern about vaccine side effects as a predictor of vaccination (21). Some studies only considered the impact of gender, residence, and previous vaccination experience on vaccination intentions and did not consider beliefs about vaccination, so these predictors should be interpreted cautiously (12). According to the knowledge attitude practice theory, knowledge and attitudes are the drivers of change in practice (22). Hence, understanding nursing students' vaccine-related knowledge and attitudes and targeting interventions is vital for reducing their COVID-19 vaccine hesitancy. This study focuses on the influence of nursing students' knowledge and attitudes on their COVID-19 vaccine hesitancy (e.g., attitudes toward vaccine safety and efficacy, perceived risk of outbreaks, perceptions of herd immunity). This study lays the foundation for further exploring and addressing the knowledge and attitudinal gaps that inform COVID-19 vaccine hesitancy. The findings provide a scientific foundation for educational, medical, and governmental organizations to develop targeted interventions to mitigate vaccine hesitancy within this population of nursing students.

## 2 Methods

### 2.1 Ethical considerations

The School of Nursing, Wannan Medical College's Ethics Review Committee approved this study (LL-202204). All participants provided informed consent.

### 2.2 Study design, setting, and participants

This study employed a cross-sectional research design and adhered to the STROBE guideline (12, 23) for reporting cross-sectional studies. The participants were recruited through a convenience sampling approach. An online survey was conducted through Wenjuanxin (www.wjx.cn) in several medical universities in Anhui Province, China, from November 9, 2022, to November 25, 2022. The researchers presented the study's purpose, significance, and privacy to the nursing students in the classroom and sent the link to the online questionnaire on social media. After completing the informed consent form, participants who volunteered for this study completed the questionnaire using an online link. A priori power analysis was conducted using G^*^Power (24) to estimate binary logistic regression analyses for detecting medium effect sizes (*f*^2^ = 0.15, OR = 3.47) (25). Two-sided test, alpha = 0.05, power (1-beta) = 0.9, estimated sample size *N* = 128.

The participants were age >18, full-time undergraduate nursing students, and informed consent to participate in this study, and individuals who could not complete the questionnaire independently or could not be vaccinated due to health problems were excluded. To enhance the quality of the questionnaire, the e-questionnaire was set up with a separate IP address, all questions were set as forced to be completed, and the survey was limited to 10 min. All questionnaires were completed in the study settings; the researchers checked them immediately to exclude invalid questionnaires (the questionnaire options were not fully completed, or the filling time was either too short or too long). A total of 1,114 questionnaires were returned, of which 1,090 were valid (97.85% validity rate).

### 2.3 Assessment and evaluation

(1) Sociodemographic characteristics, including age, sex, place of residence, recent need for an internship, recent need to go out of town, father and mother's highest education, self-assessment of physical health status, whether hepatitis B vaccine has been vaccinated in the recent 5 years, and whether adverse reactions have occurred after vaccination by other vaccines.
(2) COVID-19 Epidemic Prevention and Control Knowledge and Attitudes Questionnaire: Self-designed, including “Whether there is confidence in our country sustainable epidemic control.”; “Do you think the country is already safe from an outbreak.”; “Do you think you are safe if anyone else is vaccinated?”; “Do you worry about being infected without vaccination?”; “Whether there is a risk of infection.”
(3) COVID-19 vaccine knowledge and attitudes questionnaire: Self-designed, including “Are you worried about the vaccine's safety.”; “Are you worried about the effectiveness of the vaccine.”; “Do you agree that other protective measures can be relaxed after COVID-19 vaccination.”
(4) COVID-19 vaccine hesitancy questionnaire: The questionnaire comprises two parts. Part one, per the definition of WHO (16), a question is used to assess the degree of vaccine hesitancy among respondents. “Please indicate your willingness to be vaccinated according to your actual circumstances.” The respondent may select one of the following options: “Refuse all,” “Refuse but unsure,” “Refuse some,” “Delay,” “Accept some,” “Accept but unsure,” and “Accept all.” If the respondent who selected any of the first five options was considered to be vaccine hesitancy. Conversely, the respondent was considered willing to be vaccinated (26). Part two of the survey consisted of multiple-choice questions regarding access to information about the COVID-19 vaccine, reasons for willingness to receive the vaccine, and reasons for hesitancy to the vaccine. The complete Chinese version of the questionnaire is shown in Supplementary material.

### 2.4 Data analysis

The IBM SPSS 23.0 software was used for data analysis. The demographic characteristics of the subjects were described by frequency and percentage. Pearson's chi-square test was used to analyze the differences between the groups, and the binary logistic regression analysis was used to test the influence factors of COVID-19 vaccine hesitancy. Odds ratios and 95% confidence intervals were used to estimate associations. A two-tailed *P*-value of < 0.05 was considered significant. Finally, GraphPad Prism 9 was used to visualize the logistic regression analysis results.

## 3 Results

### 3.1 Sociodemographic characteristics of respondents

A total of 1,090 nursing students completed the survey. Details of participant characteristics are presented in Table 1. The mean age of the respondents was 20.01 ± 1.18 years; most (74.8%) of them were female, 59.4% were from rural areas, and 40.6% were from urban areas. Regarding past vaccinations, 67.33% of the respondents had been vaccinated in the past 5 years, and 10.37% of the respondents had experienced adverse reactions after vaccination.

**Table 1:** Comparison of sociodemographic characteristics for COVID-19 vaccine hesitancy (*n* = 1,090).

**Factors**	**Vaccine hesitancy**		**Vaccine acceptance**		**χ^2^**	***P***
	***n***	**%**	***n***	**%**		
**Sex**					
Male	94	34.18	181	65.82	9.439	0.002
Female	201	24.66	614	75.34		
**Are you going to internship in the near future**					
Yes	124	24.22	388	75.78	3.960	0.047
No	171	29.58	407	70.52		
**Are you going out of town in the near future**					
Yes	67	21.48	245	78.53	6.919	0.009
No	228	29.31	550	70.69		
**Father's education**					
Junior high school and below	179	20.14	533	78.56	3.849	0.050
High school and above	116	30.69	262	69.31		
**Mother's education**					
Junior high school and below	218	25.29	634	74.41	4.314	0.038
High school and above	77	32.35	161	67.65		
**Self-assessment of physical health**					
Poor	21	42.00	29	28.00	7.208	0.027
Moderate	138	27.99	355	72.01		
Good	136	24.86	411	75.14		
**Hepatitis B vaccination in recent 5 years**					
Yes	182	24.80	552	75.20	5.859	0.015
No	113	31.74	243	68.26		
**Adverse reaction to vaccination**					
Yes	51	45.13	62	54.87	20.851	< 0.001
No	244	24.97	733	75.03		
**Whether there is confidence in our country sustainable epidemic control**					
Neutral	100	48.31	107	51.69	58.427	< 0.001
Very confident	195	22.08	688	77.92		
**Do you think the country is already safe from an outbreak**					
Yes	56	62.22	34	37.78	61.43	< 0.001
No	239	23.90	761	76.10		
**Do you think you are safe if anyone else is vaccinated**					
Yes	44	57.14	33	42.86	37.974	< 0.001
No	251	24.78	762	75.22		
**Do you worry about being infected without vaccination**					
Worry	95	19.51	392	80.49	36.263	< 0.001
Neutral	134	34.36	256	65.64		
Don't worry	66	30.99	147	69.01		
**Whether there is a risk of infection**					
Yes	95	21.54	346	78.46	11.442	0.001
No	200	30.81	449	69.18		
**Do you worry about the safety of the vaccine**					
Worry	106	48.18	114	51.82	147.097	< 0.001
Neutral	141	37.70	233	62.30		
Don't worry	48	9.68	448	90.32		
**Do you worry about the effectiveness of the vaccine**					
Worry	90	45.92	106	54.02	131.086	< 0.001
Neutral	151	38.82	238	61.18		
Don't worry	54	10.69	451	89.31		
**Do you agree that other protective measures can be relaxed after COVID-19 vaccination**					
Disagree	177	23.17	587	78.83	19.561	< 0.001
Uncertainty	90	36.14	159	63.86		
Agree	28	36.36	49	63.64		

### 3.2 COVID-19 vaccine hesitancy among nursing students

This study showed that 295 (27.06%) nursing students had COVID-19 vaccine hesitancy. The main reasons for willingness to be vaccinated against COVID-19 were responding to the national promotion (84.07%), worry about COVID-19 infection (73.33%), and advice from hospitals and schools (68.15%). The main reason for the hesitancy toward the COVID-19 vaccination was the fear of the vaccine's adverse reactions (64.14%). The vaccine has just been introduced to the market, and people are worried about the unknown problems with the vaccine (62.50%). Worried about the safety of the vaccine (59.87%). Details are shown in Table 2.

**Table 2:** COVID-19 vaccination willingness of nursing students (*n* = 1,090).

**Factors**	***n***	**%**
**Reasons for willingness to vaccinate COVID-19 vaccine**^a^ **(*****n*** = **795)**		
Response to national calls	681	84.07
Fear of COVID-19 pneumonia	594	73.33
Positive guidance of hospitals and schools	552	68.15
Free universal vaccination	425	52.47
Clinical contact is required	409	50.49
Affected by the surrounding people	299	36.91
Family requirements	233	28.77
Other	37	4.57
**Reasons for hesitation in COVID-19 vaccine**^b^ **(*****n*** = **295)**		
Worried about vaccine adverse reactions	195	64.14
Vaccines have just been listed and there are uncertainties.	190	62.50
Worried about vaccine safety	182	59.87
Worried about vaccine effectiveness	134	44.08
Heard of adverse reactions to vaccination	84	27.63
Recent vaccinations	63	20.72
The inoculation needs an appointment, which is too troublesome.	46	15.13
Afraid of vaccination needle pricking pain	44	14.47
Think you 're safe, not infected	36	11.84
Nobody vaccinated around	27	8.88
I don 't want to be vaccinated, no reason	24	7.89
Someone advised me not to vaccinate	23	7.57
Other	12	3.95

^a^Vaccine acceptance = accept but unsure + accept all.

^b^Vaccine hesitancy = refuse all + refuse but unsure + refuse some + delay + accept some.

### 3.3 Comparison of sociodemographic characteristics for COVID-19 vaccine hesitancy

As shown in Table 1, several factors were identified significantly associated with COVID-19 vaccine hesitancy, including sex (*P* = 0.002), Going to the internship in the near future (*P* = 0.047), Going out of town in the near future (*P* = 0.009), Father's and mother's education (*P* = 0.050 and *P* = 0.038, respectively), Self-assessment of physical health (*P* = 0.027), Received hepatitis B vaccine in the last 5 years (*P* = 0.015), Adverse reaction to vaccination (*P* < 0.001); Confident in the ability of China to sustainably control the epidemic (*P* < 0.001); Believe that the country is safe and there will be no outbreak (*P* < 0.001); Think that as long as other people have been vaccinated, they will be safe (*P* < 0.001); Worry that they will be infected if they do not receive the vaccine (*P* < 0.001); Believe that there is a risk of infection at present (*P* = 0.001); Worry about the safety of the vaccine (*P* < 0.001); Worry about the effectiveness of the vaccine (*P* < 0.001); Agree that other protective measures can be relaxed after vaccination (*P* < 0.001).

### 3.4 Associated factors of COVID-19 vaccine hesitancy

The vaccine hesitancy was used as the dependent variable in this study (no = 0, yes = 1). Factors showing statistical differences in the Chi-square test were used as independent variables. Dummy variables were set when the independent variable was *k* ≥ 3. Due to the large number of independent variables in this study, the binary logistic regression analysis was conducted using Forward: LR (αin = 0.05, αout = 0.10), and the variables that finally entered the regression equation were eight variables, as shown in Table 3.

**Table 3:** Binary logistic regression analysis of influence factors of COVID-19 vaccine hesitancy for nursing students in China (*n* = 1,090).

**Factors**	**Comparison category**	**Reference category**	***b***	**SE**	**Wald c2**	***P***	**OR**	**95% CI for OR**
Are you going to the field in the near future.	Yes	No	−0.401	0.186	4.671	0.031	0.670	0.465–0.963
Whether there is confidence in our country sustainable epidemic control.	Very confident	Neutral	−0.933	0.182	26.359	< 0.001	0.393	0.275–0.562
Do you think the country is completely safe from an outbreak.	Yes	No	1.234	0.280	19.401	< 0.001	3.436	1.984–5.950
Do you think you are safe if anyone else is vaccinated.	Yes	No	0.806	0.310	6.749	0.009	2.239	1.219–4.113
Whether there is a risk of infection.	Yes	No	−0.419	0.165	6.467	0.011	0.658	0.476–0.908
Do you worry about the safety of the vaccine.	Don't worry	Worry	−1.488	0.266	31.419	< 0.001	0.226	0.134–0.380
	Neutral		−0.378	0.185	4.152	0.042	0.686	0.477–0.986
Do you worry about the effectiveness of the vaccine.	Don't worry	Worry	−0.889	0.232	14.701	< 0.001	0.411	0.261–0.648
Do you agree that other protective measures can be relaxed after COVID-19 vaccination.	Agree	Disagree	0.697	0.304	5.258	0.022	2.007	1.106–3.640
Constant			0.727	0.213	11.621	< 0.001	2.068	–

Likelihood ratio: χ2 = 1001.278, *P* < 0.001, Cox and Snell R2 = 0.221, Nagelker'ke R2 = 0.320. Hosmer-Lemeshow goodness of fit test: χ2 = 3.576, *P* = 0.893.

The study results showed “the need to go out of town recently” [OR = 0.670, 95% CI (0.465, 0.963)], “very confident that the outbreak could be controlled sustainably” [OR = 0.393, 95% CI (0.275, 0.562)], “feeling at risk of infection” [OR = 0.658, 95% CI (0.476, 0.908)], “not being worried/being generally worried about the vaccine's safety” [OR = 0.226, 95% CI (0.134, 0.380) and OR = 0.686, 95% CI (0.477, 0.986), respectively], and “not being worried about the vaccine's effectiveness” [OR = 0.411, 95% CI (0.261, 0.648)]. The above five factors were protective factors against COVID-19 vaccine hesitancy among nursing students (*P* < 0.05).

**Figure 1:**
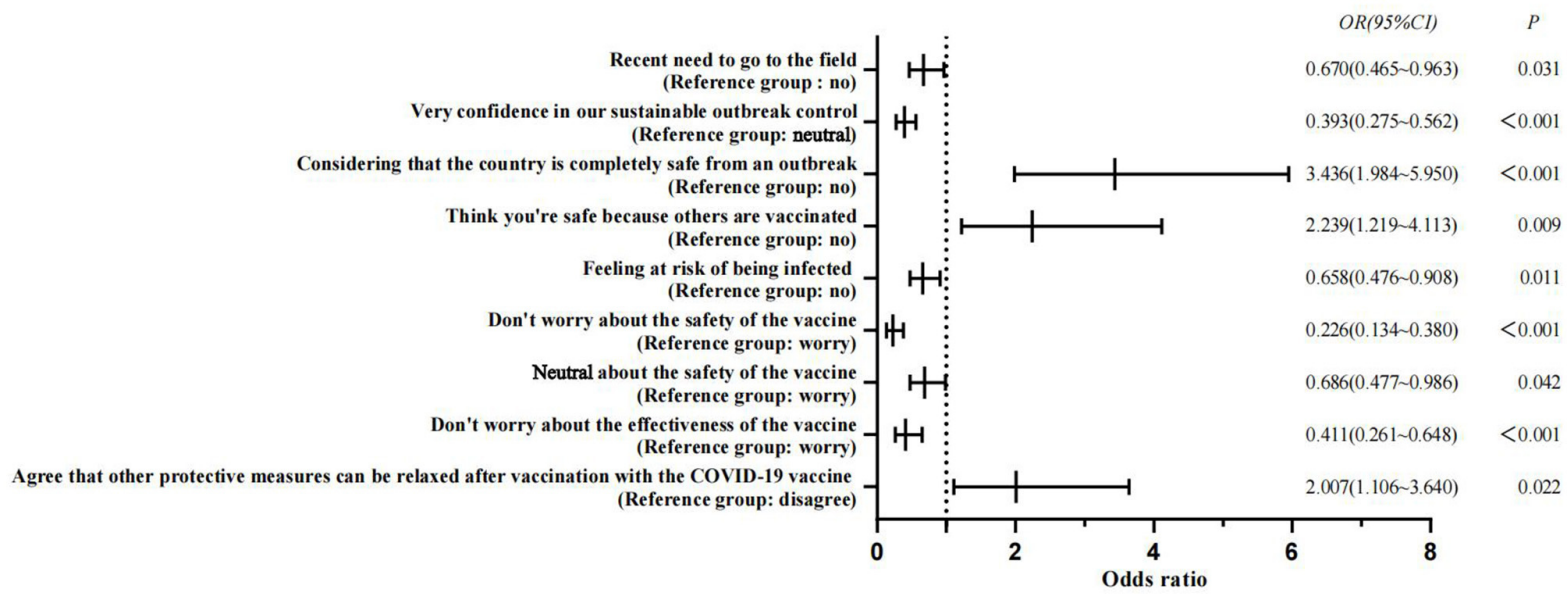
Factors associated with COVID-19 vaccine hesitancy among Chinese nursing students.

The students considering the “country completely safe from an outbreak” [OR = 3.436, 95% CI (1.984, 5.950)], “consider themselves safe because others are vaccinated” [OR = 2.239, 95% CI (1.219, 4.113)], and “agree that other protective measures can be relaxed after vaccination with the COVID-19 vaccine” [OR = 2.007, 95% CI (1.106, 3.640)] were risk factors for COVID-19 vaccine hesitancy among nursing students (*P* < 0.05). Details of the factors associated with COVID-19 vaccine hesitancy among Chinese nursing students are shown in Table 3 and Figure 1.

## 4 Discussion

The results showed that 27.06% of nursing students had COVID-19 vaccine hesitancy. A lower percentage of nursing students in this study showed vaccine hesitancy compared to the 2021 survey result (27). Various reasons may explain this outcome, such as social and school promotion; validation of vaccine safety over time, and so on. The main reason is that they are worried about adverse reactions to vaccines (64.14%), which is similar to the research results of Kin (28). The respondents' vaccine hesitancy was primarily driven by concerns about vaccine efficacy, potential adverse reactions, and the duration of protection provided by the vaccine. These concerns can be largely attributed to a lack of confidence in the vaccine and fear of its potential consequences. Furthermore, given the COVID-19 vaccine has just been introduced, there are widespread concerns about the uncertainties surrounding the COVID-19 vaccine (62.5%). This result is consistent with the study of Zhang et al. (29). The research results show that the willingness to vaccinate the COVID-19 vaccine after its emergency listing is only 42.46%. The vaccine development needs 3–5 years or even longer (30), and the public may still wait and observe the vaccine's protective effect. In addition, concerns about vaccine safety (59.87%) are one of the main reasons for vaccine hesitancy (31). Usually, vaccine side effects and adverse reactions draw the public's much attention, particularly, vaccine safety incidents that occurred during that period of time (32). In addition, studies have shown that a lack of trust in vaccination professionals and manufacturers also affects public concern about vaccine safety (33).

### 4.1 Factors associated with COVID-19 vaccine hesitancy among Chinese nursing students

A lower incidence of COVID-19 vaccine hesitancy among nursing students who recently need to go out of town [Yes vs. No, OR = 0.670, 95% CI (0.465, 0.963), *P* = 0.031] was similar to the results of Huo et al. (34). During the study period, China entered the normalization stage of epidemic prevention and control, and there were occasional aggregations caused by sporadic cases in some areas (35). Nursing students have a high degree of group mobility and aggregation, influenced by factors such as study, practice, and daily life. At the same time, some nursing students are about to enter clinical practice. Therefore, the incidence of vaccine hesitancy among nursing students was low. Comparatively, nursing students who “have to go out of town” are exposed to different environments, and populations are at higher risk of infection. Consequently, the incidence of vaccine hesitancy is lower among them.

Nursing students with high confidence in the sustainable control of the Chinese epidemic had a relatively low incidence of COVID-19 vaccine hesitancy [Very confident vs. Neutral, OR = 0.393, 95% CI (0.275, 0.562), *P* < 0.001], consistent with the findings of Cristina Giambi et al. (33). Nurses' confidence in controlling the outbreak is essentially confidence in the policy-makers. According to the “3Cs” model theory, confidence in vaccine policy-makers will promote vaccination (4). Confidence is transformed into dependence, which reduces or eliminates vaccine hesitancy. A study in Austria showed that vaccine hesitancy was 2.07–2.26 times higher among residents who expressed opposition and neutrality toward vaccine decision-makers than among those who supported vaccine policy-makers (36). Confidence is a central predictor of vaccine hesitancy, and mistrust of vaccines/government increases vaccine hesitancy (37). Similar to the results of this study, 84.07% of nursing students who were willing to receive the COVID-19 vaccine indicated that responding to the national promotion was one of the reasons they were willing to receive the vaccine. The solution to vaccine hesitancy is inseparable from the government's and medical personnel's leadership. Government agencies should continue to increase public awareness of the importance and necessity of COVID-19 vaccination.

Nursing students who “feel completely safe in the country and that there will be no outbreaks” have a higher incidence of COVID-19 vaccine hesitancy [Yes vs. No, OR = 3.436, 95% CI (1.984, 5.950), *P* < 0.001], which supports Żuk et al. (38) and Wang et al. (39) findings. Due to the stringent control measures implemented by the Chinese government to mitigate COVID-19 outbreaks during the period of 2020–2022, such as the delineation of risk zones and outbreak management strategies, the infection and mortality rates of COVID-19 in China have remained relatively low (40, 41). Consequently, some nursing students in China have not fully recognized the importance and necessity of COVID-19 vaccination for disease prevention and management, and thus do not perceive vaccination as an essential measure (42). As a result, they have become complacent about the importance of vaccination and infectious disease control (4). However, the effective management of COVID-19 outbreaks today relies on maintaining high vaccination rates, continued advancements in medical technology, and coordinated efforts by national governments (43). Vaccination remains the single most effective measure to safeguard vulnerable populations and mitigate the spread of the COVID-19 virus (44). In this regard, schools should further improve the awareness of nursing students on the susceptibility and severity of COVID-19.

The results showed that nursing students who believed they were safe as long as others were vaccinated had a higher incidence of COVID-19 vaccine hesitancy [Yes vs. No, OR = 2.239, 95% CI (1.219, 4.113), *P* = 0.009], which was aligned with the results of Pogue et al. (44). Some nursing students have misconceptions about vaccination. The main point is that “not everyone needs to be vaccinated, and group immunization is sufficient to protect everyone (44)”. Indeed, when the majority of individuals are vaccinated, the disease is prevented from spreading due to the phenomenon of herd immunity. However, those who are unvaccinated remain at a high risk of infection (45). This misconception causes some nursing students to have a wait-and-see mentality, believing that as long as others are vaccinated, they will not be infected, resulting in vaccine hesitancy. It is recommended that nursing students' epidemiological knowledge be strengthened through health education to prevent them from adopting a wait-and-see mentality.

The incidence of vaccine hesitancy among nursing students who perceived themselves to be at risk of infection was relatively low [Yes vs. No, OR = 0.658, 95% CI (0.476, 0.908), *P* = 0.011], which was similar to that of Kong (46) and Jiang and Zhang (47). Because of the nature of the medical profession, nursing students have a clearer perception of the risk of COVID-19 infection, which is one of the important reasons why nursing students choose to receive the vaccine. The study of nursing students and clinical nurses shows that the overall performance of the risk perception ability of nursing students and clinical nurses is excellent (47, 48). Higher awareness of the risk of the COVID-19 epidemic and recognition of the importance of the COVID-19 vaccine in prevention and control are the reasons for the low incidence of vaccine hesitancy among nursing students. Notably, a study conducted in the UK found that young people did not see themselves as being at risk of contracting COVID-19. This perception was driven by a lack of confidence in the government's response and a lack of belief that the vaccine could effectively prevent the disease (49).

The lower the concern about vaccine safety, the lower the incidence of vaccine hesitancy [Don't worry vs. Worry, OR = 0.226, 95% CI (0.134, 0.380), *P* < 0. 001; Neutral vs. Worry, OR = 0.686, 95% CI (0.477, 0.986), *P* = 0.042], which is comparable to the findings of Li's et al. study (50). Vaccine safety is one of the main reasons for vaccine hesitancy (51), including concerns about vaccine safety and adverse reactions. Studies have shown that vaccine problems have affected public confidence in vaccine disease prevention (32). In Japan, the incidence of COVID-19 vaccine hesitancy among women is higher, mainly due to the safety problems of human papillomavirus (HPV) vaccines in the past, which caused public and media attention to vaccine safety (52). Furthermore, considering the safety of the COVID-19 vaccine, the willingness of residents to vaccinate against COVID-19 immediately after the vaccine listing was only 23.0%, and the willingness to vaccinate against COVID-19 decreased by 35.3% (49). More people hope to delay vaccination against COVID-19 until the vaccine's safety is confirmed. The attitudes of healthcare professionals and information about vaccine safety from authoritative sources are essential to improve people's confidence in vaccine safety (53). The Internet is a major source of information about vaccines. Negative information on the Internet is one of the main reasons for suspicious vaccine safety and causes people to hesitate about vaccines (54). It is recommended that relevant institutions enhance their media supervision, implement effective network information screening management, and facilitate communication between the public and traditional institutions through Internet media to reduce vaccine-related misunderstanding.

In this study, nursing students who were not worried about vaccine effectiveness had a lower incidence of vaccine hesitancy [Don't worry vs. Worry, OR = 0.411, 95% CI (0.261, 0.648), *P* < 0.001], which was similar to the results of Pogue et al. and Wu et al. (44, 55). The respondents' vaccine hesitancy is primarily driven by concerns about its effectiveness, duration, and adverse effects. Studies have shown that, as vaccine efficacy improves, people's willingness to be vaccinated against the COVID-19 virus will increase significantly (49). It is predicted that when the efficacy of the COVID-19 vaccine reaches 90%, the willingness of the target groups to accept the vaccine will reach 45.6–73.2% in 3 months since the beginning of the vaccination plan (49). Lin and Wang (56) showed that the efficacy of the HPV vaccine and the duration of immunization were the primary determinants of HPV vaccine uptake among nursing students. In light of this, vaccine regulatory authorities must prioritize evaluating and monitoring the quality, safety, and effectiveness data associated with COVID-19 vaccines.

The findings of this study indicate that the prevalence of vaccine hesitancy was higher among nursing students who agreed to relax other protective measures after vaccination [Agree vs. Disagree, OR = 2.007, 95% CI (1.106, 3.640), *P* = 0.022], a result that is consistent with the findings reported by Liu et al. (27). The reason reflected that some nursing students were complacent about the role of the COVID-19 vaccine in preventing diseases and demonstrated misunderstandings about the vaccine. Although vaccination can produce immunization and reduce the risk of infection, its protective effect is not absolute. Some individuals may not be able to produce sufficient antibodies following vaccination, which could reduce the effectiveness of immunization. Studies have demonstrated that a lack of comprehension of vaccine characteristics and epidemics can lead to a sense of complacency (57). Similar to the results of this study, some nursing students have insufficient awareness of the characteristics of the COVID-19 vaccine and show complacency with the vaccine, resulting in vaccine hesitancy.

### 4.2 Limitations

This study had some limitations. First, it did not examine how the nursing students' perceptions of COVID-19 severity affected their vaccination intentions. The severity of illness from COVID-19 could be an important factor in their decision to get vaccinated, but the study did not address this. Additionally, the study was conducted only in Anhui Province China using a convenience sampling. This means the results may be specific to that geographic area and need to be validated in other populations. Another limitation was the cross-sectional research design, which only looked at a single point in time, rather than tracking changes over time. Vaccine hesitancy can change over time, so the researchers recommend that future studies use longitudinal tracking surveys to explore how vaccine hesitancy evolves.

## 5 Conclusion

Overall, 27.06% of participants reported COVID-19 vaccine hesitancy. The main reason for COVID-19 vaccine hesitancy is the concern about the adverse reactions of the vaccine. The vaccine has just been put on the market, and they are worried about the unknown problems of the vaccine and worried about the safety of the vaccine. Nursing students who believe that the country is completely safe from outbreaks, believe that they will not be infected as long as others are vaccinated, and agree that they can relax other protective measures after receiving the COVID-19 vaccine are at high risk for vaccine hesitancy. Schools and healthcare institutions should take relevant countermeasures to improve nursing students' understanding of the COVID-19 epidemic and the vaccine, correct their misconceptions and behaviors, improve their risk perception, and perfect their protective measures. In addition, infectious disease prevention training is needed for nursing students to reduce their vaccine hesitancy and protect their health.

## Data Availability

The raw data supporting the conclusions of this article will be made available by the authors, without undue reservation.
