# Supplementary material for: What determinants of COVID-19 vaccine hesitancy among Chinese nursing students? A cross-sectional study
Source: Front Public Health. 2024 Aug 20;12:1432225. doi: 10.3389/fpubh.2024.1432225 (PMC11368718; doi:10.3389/fpubh.2024.1432225)
Supplement: Supplementary file 1 [file Data_Sheet_1.docx]

**Appendix table 1: Chinese Nursing Students COVID-19 Vaccine Hesitancy Questionnaire (Chinese Version)**

**中国护生新型冠状病毒疫苗犹豫现状及影响因素调查**

**一、一般资料情况**

1、性别：

A.男

B.女

2、年龄：

3、生源地：

A.城市

B.农村

4、您近期是否要参与临床实习

A.是

B.否

5、您近期是否要前往外地：（跨省、市）

A.是

B.否

6、您父亲的最高学历：

A.初中及以下

B.高中

C.大专

D.本科

E.硕士及以上

7、您母亲的最高学历：

A.初中及以下

B.高中

C.大专

D.本科

E.硕士及以上

8、您自评健康状况：

A.非常差

B.比较差

C.一般

D.比较好

E.很好

9、您是否接种过乙肝疫苗（5年内）：

A.是

B.否

10、您是否有过接种其他疫苗发生不良反应的情况：

A.是

B.否

**二、新冠肺炎疫情防控知识与态度**

11、您对我国今年可以消除新型冠状病毒肺炎疫情的信心：

A.很有信心

B.一般

12、您是否觉得国内已经安全了，没必要接种疫苗：

A.是

B.否

13、您是否觉得别人都接种意愿，自己就安全了：

A.是

B.否

14、如果不接种新型冠状病毒疫苗，是否担心会感染:

A.非常担心

B.比较担心

C.不确定

D.较不担心

E.完全不担心

15、您觉得您是否有被感染的风险:

A.有

B.没有

**三、新冠疫苗知识与态度**

16、您对新型冠状病毒疫苗的安全性:

A.非常担心

B.比较担心

C.不确定

D.较不担心

E.完全不担心

17、您对新型冠状病毒疫苗的有效性:

A.非常担心

B.比较担心

C.不确定

D.较不担心

E.完全不担心

18、接种新型冠状病毒疫苗后，可以放松其他防护措施:

A.非常不同意

B.不同意

C.不确定

D.同意

E.非常同意

**四、新冠疫苗犹豫情况**

19、您对新冠疫苗的态度是：

A.拒绝所有

B.拒绝但不确定

C.有点拒绝

D.延迟接种

E.有点接受

F.接受但不确定

G.接受

20、您愿意接种新型冠状病毒疫苗的原因是：（多选题）

A.担心感染新型冠状病毒肺炎

B.医院、学校要求

C.相应国家号召

D.家人要求

E.需要接触临床

F.疫苗全民免费接种

G.受周围人影响

H.其他

21、您不愿意/不确定接种新冠疫苗的原因是：（多选题）

A.疫苗刚刚上市，有不确定因素

B.担心疫苗安全性

C.担心疫苗有效性

D.担心疫苗副作用

E.最近在接种其他疫苗

F.接种需要预约，太麻烦

G.觉得自己很安全，不会感染

H.害怕接种疫苗针扎痛

I.听说过疫苗接种不良反应

J.身边人都没接种

K.有人劝我不要接种

L.不确定
